# Patients with rheumatoid arthritis in clinical remission and ultrasound-defined active synovitis exhibit higher disease activity and increased serum levels of angiogenic biomarkers

**DOI:** 10.1186/ar4431

**Published:** 2014-01-08

**Authors:** Julio Ramírez, Virginia Ruíz-Esquide, Isaac Pomés, Raquel Celis, Andrea Cuervo, Mª Victoria Hernández, Jaume Pomés, José L Pablos, Raimon Sanmartí, Juan D Cañete

**Affiliations:** 1 Rheumatology Department, Hospital Clínic and IDIBAPS, Villarroel 170, Barcelona 08036, Spain; 2 Musculoskeletal Section, Hospital Clínic, Barcelona, Spain; 3 Rheumatology Department, Instituto de Investigación 12 de octubre, Madrid, Spain

## Abstract

**Introduction:**

The aim of this study was to identify and characterize subclinical synovitis in patients with rheumatoid arthritis (RA) in clinical remission using power Doppler ultrasound (PDUS) and serum levels of biomarkers of inflammation and/or angiogenesis.

**Methods:**

We selected patients with RA in clinical remission defined as a Disease activity score of 28 joints (DAS28)-erythrocyte sedimentation rate (ESR) <2.6 for more than six months tested by two independent rheumatologists. Clinical, epidemiological, demographic and serological data were analyzed. PDUS of knees and hands was performed by a sonographer. Synovial hypertrophy (SH) and PDUS signal were scored (grades 0 to 3). SH ≥2 and a PDUS signal was classified as active synovitis. Serum levels of biomarkers of inflammation/angiogenesis were determined by Quantibody^®^ Human Array.

**Results:**

This study included 55 patients, of whom 25 (45.4%) met criteria for ultrasound-defined active synovitis. Patients with active synovitis had higher DAS28-C reactive protein (*P* = 0.023), DAS28-ESR (*P* = 0.06), simplified disease activity score, SDAI (*P* = 0.064), and only 12% were taking oral glucocorticoids (≤5 mg/day) compared with 40% of patients without active synovitis (*P* = 0.044). Patients with synovitis also had significantly higher serum levels of the angiogenic biomarkers angiopoietin-2 (*P* = 0.038), vascular endothelial growth factor-D (*P* = 0.018), placental growth factor (*P* = 0.043), stromal cell-derived factor-1 (*P* = 0.035), matrix metallopeptidase-2 (*P* = 0.027) and basic fibroblast growth factor (bFGF) (*P* = 0.007), but not of pro-inflammatory cytokines.

In the multivariate logistic regression model used to explore prognostic biomarkers for active synovitis, serum levels of bFGF, DAS28-ESR and not receiving glucocorticoids were the best predictors of active synovitis. The predictive indexes provided by the model were specificity 73.3%, sensitivity 72%, and area under the curve in receiver operating characteristic 81.5% (95% CI: 70.1% to 92.8%).

**Conclusions:**

Nearly half of the patients with RA in clinical remission had ultrasound-defined active synovitis, higher disease activity and less frequent oral glucocorticoid consumption than patients without active synovitis. This clinical situation was associated with a specific biological profile characterized by an excess of angiogenic mediators rather than persistent proinflammatory cytokine responses.

## Introduction

Rheumatoid arthritis (RA) is a chronic inflammatory immune-mediated disease characterized by polyarticular synovitis that can lead to joint destruction with impairment of function and quality of life. Current biologic therapies and the implementation of treat-to-target strategies make remission an affordable goal. In fact, rates of remission of RA of around 50% can be achieved if intensive treatment is started in the early stages of the disease [1]. However, there is concern about whether current criteria reflect true remission characterized by the abrogation of synovitis and, consequently, a lack of radiographic progression [2].

In recent years, ultrasound and magnetic resonance imaging techniques have revealed that a significant percentage of patients classified as being in clinical remission exhibit different grades of synovitis, and a subgroup of these patients suffer flares and/or joint damage during follow-up [3-6].

One of the most conspicuous signs of synovitis is the increase in synovial vascularization due to angiogenesis, which is crucial for synovial growth and invasiveness [7]. Despite clinical improvement, the persistence of synovial vascularization, evaluated by power Doppler ultrasound (PDUS), has been associated with a higher risk of flares and joint damage [8,9].

Although studies have aimed to correlate PDUS activity in RA with cytokines implicated in angiogenesis, especially vascular endothelial growth factor (VEGF), there are no studies on the association between active synovitis as defined by PDUS and angiogenic mediators in patients with RA in clinical remission [10,11].

The objective of this study was to identify and characterize subclinical synovitis in patients with RA in clinical remission using PDUS and to characterize the biological profile of this group of patients by determining inflammatory and angiogenic biomarkers.

### Patients and methods

#### ***Patients***

Patients with RA in clinical remission for >6 months, as defined by a 28-joint Disease Activity Score (DAS28)-erythrocyte sedimentation rate (ESR) <2.6 and confirmed by two independent rheumatologists, were consecutively selected from our Arthritis Unit outpatient clinic. Clinical, demographic and serological data were collected, including low-dose oral prednisone treatment, disease-modifying antirheumatic drugs and biological therapy. Rheumatoid factor (RF) was determined by nephelometry and anti-citrullinated peptide/protein antibodies (ACPA) by anti-cyclic citrullinated peptide enzyme-linked immunosorbent assay kits (Immunoscan; Eurodiagnostica, Malmö, Sweden – distributed by Diasorin, Madrid, Spain). Signed inform consent was obtained from all patients. The study was approved by the Ethics Committee of the Hospital Clinic.

## Methods

All sonographic assessments were performed using high-sensitivity ultrasound equipment (Acuson Antares^®^; Siemens AG, Erlangen, Germany). Sonographic assessments were performed using a frequency range from 8 to 12 MHz.

Joint ultrasound findings were defined according to published OMERACT definitions [12]. The frequency was adapted to each joint assessed. A high frequency (12 MHz) was used in superficial joints such as the metacarpophalangeal (MCP) or the proximal interphalangeal, and a lower frequency (8 to 10 MHz) was used in the knees.

An experienced sonographer who was unaware of the results of the clinical joint examination evaluated both knees and 11 joints of each hand (including the proximal interphalangeal joints, the MCP joints and the wrists) for both synovial hypertrophy (SH) and intra-articular PDUS signals according to EULAR guidelines [13]. All joints evaluated were scanned for SH and PDUS on the dorsal aspect (except for the knee, where the suprapatellar and lateral and medial parapatellar recesses were evaluated in the anterior aspect with the knee in extension) using longitudinal midline and transversal planes. The wrists were additionally examined using longitudinal dorso-radial and dorso-ulnar scans.

Synovial PDUS was assessed by selecting a region of interest that included the bony margins, joint space and a variable view of surrounding tissues. PDUS calibrations were adjusted at the lowest permissible pulse-repetition frequency to maximize sensitivity (500 to 800 Hz). The Doppler frequency was set higher for the study of small joints and superficial tissues, and lower for deep structures like the knee. Color gain was set just below the level that causes the appearance of noise artifacts. The sonographer was allowed to modify the machine setting (for example, gain, pulse-repetition frequency) in order to produce the best quality images, allowing each image to be scored appropriately.

SH and PDUS were graded using a four-grade semiquantitative scoring system from 0 to 3 (grade 0 = no, 1 = mild, 2 = moderate and 3 = severe) according to the method developed by Szkudlarek and colleagues [14]. The highest SH and PDUS grade detected during the scans was adopted as representative of each joint, respectively. This method was adapted to the knee ultrasound assessment (SH: grade 0 = no; 1 = mild, flat thickened synovium; 2 = moderate, thickened synovium with few villi-like protrusions; 3 = severe, marked thickening with multiple villi-like protrusions; and PDUS: grade 0 = no flow in the synovium, 1 = single vessel signals, 2 = confluent vessel signals in less than half the area of the synovium, 3 = vessel signals in more than half the area of the synovium).

**Figure 1:**
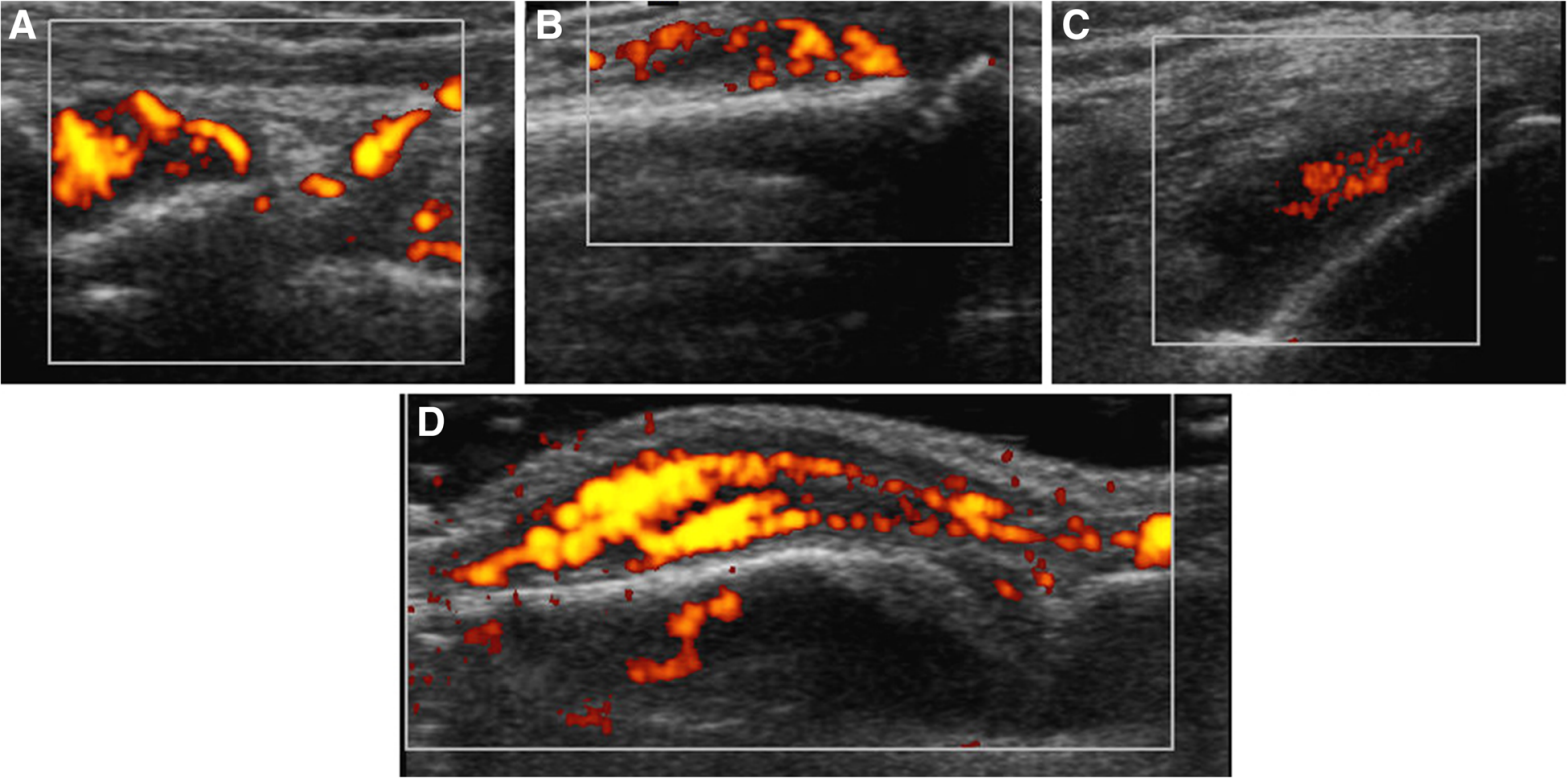
**Representative images of ultrasound-defined synovitis in assessed joints. (A)** Radiocarpal joint, longitudinal view: synovial hypertrophy (SH) grade 2 and power Doppler ultrasound (PDUS) signal grade 3. **(B)** Proximal interphalangeal joint, longitudinal view: SH grade 2 and PDUS signal grade 2. **(C)** Knee joint: parapatellar recess, transversal view: SH grade 2 and PDUS signal grade 2. **(D)** Metacarpophalangeal joint, longitudinal view: SH grade 2 and PDUS signal grade 3.

To ensure a stringent definition of synovitis by ultrasound, only patients with SH grade ≥2 plus PDUS signal were classified as having active synovitis. If any assessed joint met these criteria, the patient was classified as having active synovitis. Examples of joints fulfilling these criteria are shown in Figure 1.

Intra-rater agreement was 0.81 for SH and 0.92 for PDUS. We made a double ultrasound assessment in the first 10 patients included in the study. The two evaluations were separated by between 24 and 72 hours. The same sonographer made both ultrasound explorations and noted the results. This index was calculated as the percentage of agreement between these scores at two time points. The following cutoff values, analogous to kappa coefficients, were defined for intra-rater reliability: <0.0 = none, 0 to 0.20 = poor, 0.21 to 0.40 = modest, 0.41 to 0.60 = fair, 0.61 to 0.80 = good and 0.81 to 1.00 = excellent.

### Quantification of biomarkers of inflammation/angiogenesis

Cytokines and angiogenic mediators were analyzed using Quantibody^®^ Human Custom Array (RayBiotech, Norcross, GA, USA), which includes: activin A, angiopoietin (ANG), ANG-1, ANG-2, angiostatin, angiopoietin-like protein-4 (ANGPTL4), basic fibroblast growth factor (bFGF), transforming growth factor beta-1, placental growth factor (PlGF), VEGF, VEGF-D, vascular endothelial growth factor receptor (VEGFR)-1, VEGFR-2, tyrosine kinase with immunoglobulin-like and endothelial growth factor-like domains-1 (Tie-1), tyrosine-kinase with immunoglobulin-like and EGF-like domains-2 (Tie-2), epithelial cell-derived neutrophil-activating peptide-78 (ENA-78), Growth Related Oncogene (GRO), stromal-cell derived factor-1 (SDF-1), CC-chemokine ligand (CXCL)-16, tumor necrosis factor alpha (TNFα), interleukin (IL)-6, IL-8, IL-17A, IL-17 F, IL-18, IL-20, IL-23, IL-33, matrix metalloproteinase (MMP)-2, and MMP-9, according to the manufacturer’s specifications. Each sample was diluted twofold and prepared in quadruplicate. An Axon scanner 4000B with GenePix software (Molecular Devices, Sunnyvalley, California, USA) was used to collect fluorescence intensities. Detection limits for cytokines are displayed on the manufacturer’s website [15]. After sample dilution, the effect of RF on the final results was estimated to be around 1% [16].

### Statistical analysis

Clinical variables, biomarkers and rates of therapeutic drugs were compared between patients with and without synovitis. The analysis was performed using the Mann–Whitney test and 95% median confidence interval (CI; Hodges–Lehmann) or the chi-square test, Fisher’s exact test and relative risk estimation with 95% CIs. Associations between biomarker concentrations and other clinical variables were studied using Spearman’s nonparametric correlation and the Mann–Whitney test.

Prognostic factors for synovitis were analyzed in a multivariate logistic regression model. Biomarkers were transformed using the binary logarithm. Variables with *P* ≤0.2 in the univariate analysis (Spearman’s correlation) were included in the multivariate analysis. The best predictive model was selected using R library ‘glmulti’ [17]. The final model selected had the lowest Akaike information criteria. The odds ratios and 95% CIs, *P* values for selected prognostic factors, model *P* values, MC Fadden *R*^2^, specificity, sensibility, predictive values and the area under the receiver operating curve from the final model were calculated.

For all tests, *P* ≤0.05 was considered significant. The statistical analysis was made using the R statistical program, version 3.0 [17].

## Results

### Clinical, demographic and serological characteristics

Fifty-five patients with RA in remission (76% female) aged (median) 52 years were included; disease duration at inclusion was 90 months and remission duration was 37 months; 71% were RF-positive and 86% were ACPA-positive; C-reactive protein (CRP) was 0.10 mg/dl, ESR was 9, DAS28-ESR was 2.03, DAS28-CRP was 1.42, Simplified Disease Activity Index was 4.5, and modified Health Assessment Questionnaire score was 0.1 (see Table 1).

**Table 1:** Clinical, demographic and serologic data of 55 patients with and without synovitis

	**All (*n*** **= 55)**	**Ultrasound-defined active synovitis**		***P *** **value**^**a**^
		**Yes (*n*** **= 25)**	**No (*n*** **= 30)**	
Age (years)	52.0 (44.0 to 61.5)	51.0 (44.0 to 59.0)	55.0 (47.0 to 63.3)	0.279
Sex (male)	13 (23.6%)	8 (32.0%)	5 (16.7%)	0.311
Body mass index	25.2 (23.2 to 28.3)	26.0 (24.0 to 29.0)	24.0 (23.1 to 27.4)	0.122
Disease duration (months)	90.0 (57.1 to 148.9)	86.3 (60.3 to 153.8)	93.7 (54.9 to 145.2)	0.659
Remission duration (months)	37 (8 to 58)	29 (7.50 to 50.00)	37 (9.00 to 66.25)	0.537
DAS28-ESR	2.03 (1.67 to 2.44)	2.24 (1.94 to 2.55)	1.92 (1.56 to 2.16)	*0.060*
DAS28-CRP	1.42 (1.38 to 1.58)	1.54 (1.39 to 1.67)	1.40 (1.23 to 1.48)	**0.023**
SDAI	4.51 (3.56 to 6.21)	5.03 (4.11 to 7.70)	4.14 (2.69 to 6.02)	*0.064*
Patient global assessment	30 (10 to 30)	30 (10 to 30)	30 (10 to 30)	0.767
Physician global assessment	10 (10 to 30)	10 (10 to 30)	10 (10 to 30)	0.322
VAS pain	9 (2 to 25)	14 (4 to 23)	5 (2 to 25)	0.251
VAS fatigue	15 (2 to 47)	14 (0 to 43)	16 (3 to 57)	0.529
mHAQ	0.1 (0.0 to 0.3)	0.1 (0.0 to 0.3)	0.0 (0.0 to 0.3)	0.570
ESR (mm/1 hour)	9 (7 to 15)	10 (7 to 20)	9 (6 to 12)	0.498
CRP (mg/dl)	0.10 (0.03 to 0.33)	0.11 (0.03 to 0.49)	0.09 (0.04 to 0.23)	0.728
Rheumatoid factor	39 (71%)	18 (72%)	21 (70%)	0.900
Rheumatoid factor (IU)	103 (22 to 229)	84 (22 to 240)	113 (23 to 215)	0.980
ACPA	47 (86%)	23 (92%)	24 (80%)	0.383
ACPA titers (IU/ml)	254 (109 to 1575)	270 (121 to 1352)	226 (83 to 1600)	0.645
Prednisone	15 (27%)	3 (12%)	12 (40%)	**0.044**
DMARDs	45 (82%)	22 (88%)	23 (77%)	0.463
Biological therapy	23 (42%)	9 (36%)	14 (47%')	0.600
SH (grade ≥2)	31 (56%)	25 (100%)	6 (20%)	**<0.001**
PDUS	35 (64%)	25 (100%)	10 (33%)	**<0.001**

Data expressed as median (interquartile range) or as number (percentage). ACPA, anti-cyclic citrullinated peptide/protein antibody; CRP, C-reactive protein; DAS28, 28-joint Disease Activity Score; DMARD, disease-modifying antirheumatic drug; ESR, erythrocyte sedimentation rate; mHAQ. Modified Health Assessment Questionnaire; PDUS, power Doppler ultrasound; SDAI, Simplified Disease Activity Index; SH, synovial hypertrophy; VAS, visual analog scale. ^a^Mann–Whitney test.

Fifteen (27%) patients were taking low-dose oral prednisone (dose ≤5 mg /day), 45 (82%) patients disease-modifying antirheumatic drugs (95% methotrexate), and 23 (42%) patients biological therapies (Table 1).

### Power Doppler ultrasound findings

Eighty-nine percent of the patients had SH (grade 1 included) in at least at one joint, and 64% had a PDUS signal, mainly in the wrist (34.4% right; 32.7% left), second MCP (8.6% right; 6.8 left) and knees (5.1% right; 8.6% left).

### Higher disease activity and no low-dose oral prednisone treatment in patients with ultrasound-defined active synovitis

Twenty-five (45.4%) patients met the criteria for ultrasound-defined active synovitis. These patients had higher DAS28-CRP (*P* = 0.023), DAS28-ESR (*P* = 0.06) and Simplified Disease Activity Index (*P* = 0.064) scores. A significantly lower percentage of patients with active synovitis were in treatment with oral steroids (≤5 mg/day) compared with patients without active synovitis (*P* = 0.044). No differences in remission duration or in other clinical, biological, serological or treatment differences were found between groups (Table 1).

### Ultrasound-defined active synovitis is associated with higher serum levels of angiogenic biomarkers

**Figure 2:**
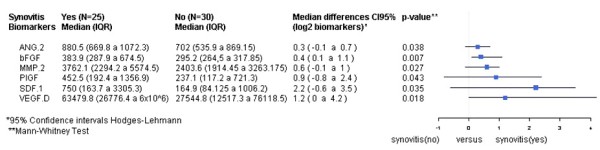
**Serum biomarkers differentially expressed in patients with and without ultrasound-defined synovitis.** ANG, angiopoietin; bFGF, basic fibroblast growth factor; MMP, matrix metalloproteinase; PlGF, placental growth factor; SDF-1, stromal-cell derived factor-1; VEGF, vascular endothelial growth factor. CI, confidence interval; IQR, interquartile range.

Patients with active synovitis had significantly higher serum levels of angiogenic biomarkers thought to be relevant to RA pathogenesis, including VEGF-D (*P* = 0.018), ANG-2 (*P* = 0.038), PlGF (*P* = 0.043), SDF-1 (*P* = 0.035), MMP-2 (*P* = 0.027) and bFGF (*P* = 0.007) (Table 2 and Figure 2). However, no association or correlations were found between active synovitis and proinflammatory cytokines (TNFα, IL-6, IL-8, IL-17A, IL-17 F, IL-18, IL-20, IL-23, and IL-33) (Table 3).

**Table 2:** Biomarkers in patients with and without ultrasound-defined synovitis

	**All (*n*** **= 55)**	**Ultrasound-defined active synovitis**		***P *** **value**^**a**^
		**Yes (*n*** **= 25)**	**No (*n*** **= 30)**	
Activin A	6,556 (3,012 to 10,887)	9,186 (4,846 to 12,297)	4,175 (2,450 to 10,255)	0.086
ANG	7,670 (6,206 to 10,496)	8,009 (6,729 to 10,816)	6867 (5,861 to 9,421)	0.119
ANG-1	32,622 (28,287 to 36,705)	34,343 (28,615 to 39,515)	31,066 (27,977 to 35,834)	0.210
ANG-2	726.4 (595.8-1014.8)	881 (670 to 1,072)	702 (536 to 869)	**0.038**
Angiostatin	37,409 (30,333 to 48,183)	34,326 (28,123 to 46,416)	39,648 (34,243 to 49,152)	0.111
ANGPTL4	15,120 (6,450 to 6,464)	1,374 (606 to 6,030)	2,294 (7,450 to 7,713)	0.630
bFGF	314 (268 to 492)	384 (288 to 675)	295 (265 to 318)	**0.007**
CXCL16	849 (739 to 1,311)	907 (774 to 1,214)	848 (682 to 1,454)	0.967
ENA-78	1,186 (813 to 1,746)	1,146 (871 to 1,664)	1,241 (780 to 1,886)	0.927
GRO	9,570 (7,592 to 12,000)	10,724 (8,644 to 12,000)	9,284 (7,050 to 12,000)	0.350
IL-17	10 (0 to 27)	13 (5 to 32)	7 (0 to 25)	0.141
IL-17 F	288 (57 to 4,888)	344 (59 to 3,351)	204 (58 to 4,980)	0.872
IL-18	589 (395 to 1,066)	741 (463 to 1,224)	514 (341 to 890)	0.066
IL-20	259 (92 to 836)	422 (145 to 924)	234 (68 to 727)	0.098
IL-23	249 (176 to 431)	257 (219 to 699)	240 (166 to 323)	0.098
IL-33	14 (4 to 73)	17 (4 to 69)	11 (4 to 83)	0.866
IL-6	149 (93 to 290)	177 (106 to 317)	136 (91 to 226)	0.136
IL-8	30 (21 to 62)	42 (21 to 64)	26 (22 to 46)	0.366
MMP-2	2,738 (1,941 to 4,050)	3,762 (2,294 to 5,574)	2,404 (1,915 to 3,263)	**0.027**
MMP-9	28,180 (21,104 to 34,808)	27,370 (23,526 to 34,009)	29,008 (17,682 to 35,779)	0.940
PlGF	288 (148 to 979)	453 (192 to 1,357)	237 (117 to 721)	**0.043**
SDF-1	280 (117 to 1,554)	750 (164 to 3,305)	165 (84 to 1,006)	**0.035**
TGF-β1	1,453 (809 to 2,252)	1,741 (1,065 to 2,424)	1,196 (649 to 2,053)	0.089
Tie-1	5,099 (1,647 to 19,737)	6,841 (1,896 to 31,161)	4,039 (1,556 to 12,210)	0.112
Tie-2	14,735 (6,215 to 32,401)	20,559 (7,426 to 47,483)	11,436 (4,101 to 26,744)	0.317
TNF**α**	6,755 (4,460 to 12,112)	6,755 (4,539 to 12,869)	6,362 (4,379 to 10,507)	0.465
VEGF	1,193 (815 to 1,990)	1,193 (871 to 2,178)	1,193 (810 to 1,911)	0.609
VEGFR-1	485 (308 to 881)	634 (366 to 1,001)	471 (278 to 723)	0.204
VEGFR-2	1,014 (713 to 1,918)	1,096 (869 to 1,807)	1,005 (679 to 1,937)	0.149
VEGF-D	32,097 (17,815 to 227,618)	63,480 (26,776 to 600,000)	27,545 (12,517 to 76,119)	**0.018**

Data expressed as median (interquartile range). ANG, angiopoietin; ANGPTL4, angiopoietin-like protein-4; bFGF, basic fibroblast growth factor; CXCL, CC-chemokine ligand; ENA-78, epithelial cell-derived neutrophil-activating peptide-78; GRO, growth-related oncogene; IL, interleukin; MMP, matrix metalloproteinase; PlGF, placental growth factor; SDF-1, stromal-cell derived factor-1; TGF-β1, transforming growth factor beta-1; Tie, tyrosine-kinase with immunoglobulin-like and endothelial growth factor-like domains; TNFα, tumor necrosis factor alpha; VEGF, vascular endothelial growth factor; VEGFR, vascular endothelial growth factor receptor. ^a^Mann–Whitney test.

**Table 3:** Correlation between clinical, biological and ultrasound data and serum biomarkers

	**Ultrasound-defined synovitis**		**SH ≥2**		**PDUS ≥1**		**ACPA titers**		**RF titers**		**CRP**		**DAS28-ESR**		**DAS28-CRP**	
	***R***^**2**^	***P *** **value**^**a**^	***R***^**2**^	***P *** **value**^**a**^	***R***^**2**^	***P *** **value**^**a**^	***R***^**2**^	***P *** **value**^**a**^	***R***^**2**^	***P *** **value**^**a**^	***R***^**2**^	***P *** **value**^**a**^	***R***^**2**^	***P *** **value**^**a**^	***R***^**2**^	***P *** **value**^**a**^
Activin A	0.234	0.085	0.228	0.093	0.164	0.231	**0.409**	**0.002**	-0.000	0.973	0.183	0.181	0.095	0.486	0.199	0.144
ANG	0.213	0.117	0.214	0.115	0.150	0.274	**0.275**	**0.042**	0.157	0.250	0.078	0.567	0.054	0.691	**0.273**	**0.044**
ANG-2	**0.283**	**0.036**	0.214	0.115	0.059	0.666	0.078	0.567	-0.130	0.314	0.081	0.553	0.001	0.989	0.189	0.165
ANG	-0.210	0.109	-0.090	0.513	**-0.295**	**0.029**	-0.010	0.908	**0.342**	**0.011**	0.083	0.544	0.043	0.751	-0.250	0.064
ANGPTL4	-0.060	0.628	-0.010	0.920	-0.110	0.387	0.116	0.398	**0.268**	**0.048**	**0.352**	**0.008**	-0.190	0.160	-0.050	0.714
bFGF	**0.366**	**0.006**	*0.265*	*0.050*	*0.264*	*0.051*	**0.335**	**0.012**	1.000	0.094	0.096	0.485	-0.030	0.825	0.186	0.173
CXCL16	0.006	0.960	-0.040	0.738	0,066	0,629	0.251	0.064	-0.030	0.787	**0.283**	**0.036**	0.003	0.978	0.067	0.626
ENA-78	0.013	0.920	-0.060	0.639	0,019	0,890	0.164	0.231	0.083	0.544	0.035	0.795	**0.400**	**0.002**	0.000	0.968
GRO	0.128	0.350	0.079	0.565	-0,010	0,888	**0.341**	**0.011**	0.090	0.510	0.054	0.693	0.140	0.308	0.064	0.641
IL-17 F	0.023	0.868	0.050	0.713	0,042	0,756	0.206	0.130	0.083	0.573	**0.358**	**0.007**	-0.090	0.502	0.098	0.474
IL-18	0.250	0.065	0.154	0.259	0,188	0,169	**0.323**	**0.016**	**0.537**	**<0.001**^**b**^	0.190	0.163	-0.080	0.528	0.106	0.441
IL-20	0.227	0.095	0.193	0.156	0,133	0,332	**0.409**	**0.002**	**0.306**	**0.023**	0.097	0.481	0.049	0.719	0.195	0.153
IL-33	0.027	0.841	0.076	0.580	0,014	0,918	**0.291**	**0.031**	**0.310**	**0.021**	**0.323**	**0.016**	-0.160	0.220	0.107	0.433
MMP-2	**0.301**	**0.025**	**0.268**	**0.048**	0,178	0,192	**0.368**	**0.006**	**0.454**	**<0.001**^**b**^	-0.000	0.988	0.002	0.987	0.166	0.225
PlGF	**0.276**	**0.041**	0.207	0.128	0,202	0,138	**0.354**	**0.008**	**0.535**	**<0.001**^**b**^	0.162	0.237	0.106	0.439	0.207	0.128
SDF-1	**0.288**	**0.033**	0.212	0.119	0,214	0,116	**0.359**	**0.007**	0.167	0.222	0.144	0.294	0.074	0.587	0.178	0.193
TGF-β1	0.232	0.088	**0.298**	**0.027**	0,085	0,534	0.042	0.758	*0.263*	*0.052*	0.217	0.110	-0.100	0.443	0.211	0.121
Tie-1	0.217	0.111	0.224	0.100	0,070	0,610	**0.439**	**0.001**	**0.574**	**<0.001**^**b**^	0.130	0.343	0.026	0.848	0.209	0.125
Tie-2	0.138	0.315	0.191	0.161	0,011	0,931	**0.418**	**0.001**	**0.396**	**0.003**	0.129	0.345	0.035	0.796	0.168	0.219
TNFα	0.101	0.462	0.163	0.232	-0,030	0,809	*0.265*	*0.050*	**0.514**	**<0.001**^**b**^	0.124	0.365	-0.040	0.750	0.078	0.569
VEGF	0.071	0.605	0.039	0.776	0,133	0,332	0.114	0.405	**0.480**	**<0.001**^**b**^	0.099	0.470	**-0.288**	**0.033**	-0.200	0.129
VEGFR-1	0.174	0.202	0.219	0.108	0,078	0,569	**0.367**	**0.006**	**0.594**	**<0.001**^**b**^	0.083	0.547	0.151	0.270	**0.266**	**0.049**
VEGF-D	**0.324**	**0.016**	0.239	0.078	0,157	0,251	**0.327**	**0.015**	*0.264*	*0.051*	0.118	0.388	-0.000	0.954	0.100	0.467

^a^Spearman’s nonparametric correlation. ^b^*P* <0.01 adjusted for multiple testing (Bonferroni). ACPA, anti-cyclic citrullinated peptide/protein antibody; ANG, angiopoietin; ANGPTL4, angiopoietin-like protein-4; bFGF, basic fibroblast growth factor; CXCL, CC-chemokine ligand; CRP, C-reactive protein; DAS28, 28-joint Disease Activity Score; ENA-78, epithelial cell-derived neutrophil-activating peptide-78; ESR, erythrocyte sedimentation rate; GRO, growth-related oncogene; IL, interleukin; MMP, matrix metalloproteinase; PDUS, power Doppler ultrasound; PlGF, placental growth factor; RF, rheumatoid factor; SDF-1, stromal-cell derived factor-1; SH, synovial hypertrophy; TGF-β1, transforming growth factor beta-1; Tie, tyrosine-kinase with immunoglobulin-like and endothelial growth factor-like domains; TNFα, tumor necrosis factor alpha; VEGF, vascular endothelial growth factor; VEGFR, vascular endothelial growth factor receptor.

### Correlations between biomarkers of inflammation/angiogenesis and other clinical and sonographic variables

#### ***Ultrasound variables***

SH grade ≥2 correlated with MMP-2 and transforming growth factor beta-1 and showed a strong trend to correlation with bFGF levels (Table 3). Interestingly, all these mediators can be produced by synovial stromal cells and infiltrating leukocytes, whereas a PDUS signal only correlated, and negatively, with levels of angiostatin, an inhibitor of angiogenesis. In addition, a strong trend to correlation between the PDUS signal and bFGF was found. These results suggest that bFGF is the only angiogenic mediator differentially expressed both in relevant synovial hypertrophy grade >2 and in synovial membrane with positive PDUS (Table 3).

#### ***Inflammation and disease activity***

CRP levels strongly correlated with ANGPTL4, chemokines (CXCL16) and several proinflammatory cytokines (IL-17 F and IL-33), whereas disease activity correlated with epithelial cell-derived neutrophil-activating peptide-78, ANG and VEGFR-1. Globally, these findings reflect the expected correlation between systemic markers of inflammation and proinflammatory cytokines levels (Table 3).

#### ***Autoantibodies***

ACPA and RF titers strongly correlated with serum levels of several biomarkers (Table 3). When only RF-negative patients were analyzed (*n* = 16), the correlations between ACPA titers (*n* = 11) and serum levels of angiogenic factors were maintained. Moreover, RF-negative patients with ultrasound-defined active synovitis had significantly higher serum levels of several angiogenic factors than patients without active synovitis (data not shown).

### Basic fibroblast growth factor, no low-dose oral prednisone and DAS28-ESR as predictors of ultrasound-defined active synovitis in RA patients in clinical remission

**Figure 3:**
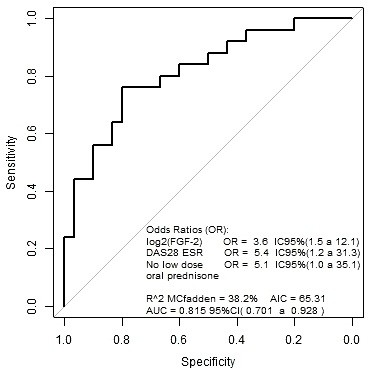
**Receiving operating curve from the model (basic fibroblast growth factor + Disease Activity Score in 28 joints + no oral prednisone treatment).** AIC, Akaike information criteria; AUC, area under the receiver operating curve; CI, confidence interval; DAS28, 28-joint Disease Activity Score; ESR, erythrocyte sedimentation rate; FGF, fibroblast growth factor.

The multivariate logistic regression model designed to explore prognostic biomarkers for active synovitis included variables with *P* ≤0.2 in the univariate analysis. Owing to the strong correlation found between RF and several serum biomarkers, RF titers were also included in the model. After excluding any significant effect of RF, higher serum bFGF, higher DAS28-ESR scores and no low-dose oral prednisone were the best predictors of active synovitis. Log_2_ bFGF, DAS28-ESR and no low-dose oral prednisone adjusted odds ratio were 3.6 (95% CI: 1.5 to 12.1), 5.4 (95% CI: 1.3 to 31.3) and 5.1 (95% CI: 1.0 to 35.1), respectively. The predictive indexes provided by the model were: specificity 73.3%, sensitivity 72%, positive predictive value 69.2%, negative predictive value 75.9% and area under the receiver operating curve 81.5% (95% CI: 70.1 to 92.8%) (Figure 3).

## Discussion

This study shows that nearly one-half of our patients with RA in clinical remission, as defined by stringent clinical criteria, had signs of active synovitis (SH grade ≥ 2 plus PDUS signal). Clinically, these patients had significantly higher disease activity and fewer were taking oral glucocorticoids compared with patients with no criteria of active synovitis. Although there were no differences in acute phase reactants between groups, patients with active synovitis had significantly higher serum levels of several angiogenic factors thought to be relevant to RA pathogenesis. A predictive index was developed for serum levels of log_2_ bFGF, no low-dose oral prednisone treatment and DAS28-ESR with an area under the receiver operating curve of 0.815.

Studies have shown that more than 40% of RA patients in clinical remission exhibit an increased PDUS signal, which could explain why a proportion of these patients develop radiographic progression during follow-up [3,5]. PDUS has been shown to add value to the clinical examination both in improving the early diagnosis of RA and in establishing true RA remission. SH scored by gray-scale ultrasound seems less specific, with grade 1 being a frequent finding in healthy controls [18]. Although there is currently no clear definition of active synovitis on ultrasound, experts suggest including SH plus PDUS signal in its definition [19]. We therefore defined active synovitis as SH grade ≥2 with PDUS signal, which may be sufficiently specific to capture only patients with potentially clinically relevant synovitis. Currently there is no universally accepted combination of joints that should be included in the ultrasound assessment of RA patients in remission [20], and therefore we followed the recommendations of Filer and colleagues [21] that suggest at least the wrist and MCP joints of the dominant hand should be assessed. To increase the sensitivity, we scored both hands (including the wrist, MCP and proximal interphalangeal joints) and knees.

Significantly fewer patients with ultrasound-defined active synovitis were taking low-dose oral prednisone than patients without active synovitis, confirming the results of other studies that found low-dose oral steroids lead to a greater probability of clinical remission and PDUS negativity at 1 year of follow-up in a treat-to-target protocol in early-onset RA patients [22]. The question therefore arises as to whether low-dose oral prednisone should be added for patients in clinical remission with ultrasound-defined active synovitis [23].

To our knowledge, no studies have assessed differences in serum biomarkers of inflammation and disease activity in patients with RA in clinical remission with and without ultrasound-defined active synovitis. Using stringent ultrasonographic criteria, we identified a significant subgroup of patients with subclinical activity. These patients were characterized by higher DAS28 and increased serum levels of several angiogenic factors (ANG-2, VEGF-D, PlGF, SDF-1, bFGF and MMP-2) but there were no differences in proinflammatory cytokines (TNFα, IL-6, IL-8, IL-17A, IL-17 F, IL-18, IL-20, IL-23, IL-33). A model including serum levels of bFGF, no low-dose oral prednisone treatment and DAS28-ESR performed well in predicting active ultrasonographic synovitis, with the predictive value being independent of RF. These data suggest that different mechanisms are involved in clinical and subclinical synovitis. An enhanced angiogenic response rather than proinflammatory cytokines or systemic inflammation biomarkers (CRP, ESR) might underlie subclinical synovitis.

Other correlations between biomarkers of inflammation/angiogenesis and SH, PDUS signal, ACPA or RF titers, DAS28 and CRP were found, notably a strong positive correlation between RF and ACPA titers and serum levels of several biomarkers. However, the multivariate logistic regression model showed that the association between angiogenic biomarkers and active synovitis was not influenced by RF. Likewise, we found no significant differences between the prevalence or titers of RF and ACPA in patients with or without active synovitis, and higher levels of angiogenic biomarkers in seronegative patients with active synovitis. There was also a significant correlation between ACPA titers and angiogenic biomarkers in seronegative patients.

The strong correlation between some proinflammatory cytokines (IL-17 F and IL-33) and CRP levels gives biological consistency to our results. Interestingly, all mediators that were significantly increased in patients with ultrasound-defined active synovitis have previously been implicated in the pathogenesis of arthritis, mainly in angiogenesis and inflammation, which also gives physiopathological relevance to these findings. ANG-2 is expressed in chronic synovitis, and is associated with increased proliferation of synovial vessels that is reversed by anti-TNF therapy [24]. PlGF is a specific ligand for VEGFR-1 and induces the growth and migration of endothelial cells. PlGF is highly expressed in the synovial tissue and fluid of RA patients and its primary source is fibroblast-like synoviocytes [25]. SDF-1 (CXCL12) is a chemokine extensively expressed by stromal cells in the synovial membrane in RA and plays a role in angiogenesis [26]. Finally, bFGF is mitogenic for endothelial cells and its overexpression worsens inflammation and joint damage in antigen-induced arthritis models in rats. However, experimental work suggests that bFGF may contribute to arthritis by increasing synovial angiogenesis, rather than through any direct effect on inflammation [27].

bFGF was the biomarker that best identified US-defined active synovitis, as it was also the only biomarker with higher, almost significant, differences in active synovitis both in SH grade ≥2 and in the PDUS signal, suggesting that it may be a good biomarker of stromal cell activity. We previously found that synovial stromal cells, fibroblast-like synoviocytes and mature vessels remain overrepresented in patients with a good clinical response to TNFα inhibitors [28,29]. However, whether there is any direct relationship between synovial stromal cells and the angiogenic biomarkers we detected remains to be determined.

This study has some limitations. The sample size and the cross-sectional design limit the strength of the conclusions. In addition, ultrasound scoring of the knee joint and ultrasound-defined active synovitis are not validated. However, we believe that the findings of this exploratory study provide new insights into the concept of clinical remission in RA and open up new avenues in the search for biomarkers that may be useful in the follow-up of RA patients in clinical remission.

## Conclusion

This study found that 45% of RA patients with stringent clinical remission criteria had ultrasound-defined active synovitis. This group of patients had a higher DAS28 and fewer were taking oral prednisone compared with patients without synovitis. These patients had significantly higher levels of several angiogenic biomarkers, especially bFGF, which correlated with different clinical and serological variables. These findings suggest it may be possible to find surrogate serum biomarkers of active synovitis that could be useful in the follow-up of patients with RA in remission.

## Abbreviations

ACPA: anti-citrullinated peptide/protein antibodies; ANG: angiopoietin; ANGPTL4: angiopoietin-like protein-4; bFGF: basic fibroblast growth factor; CI: confidence interval; CRP: C-reactive protein; CXCL: CC-chemokine ligand; DAS28: 28-joint Disease Activity Score; ESR: erythrocyte sedimentation rate; IL: interleukin; MCP: metacarpophalangeal; MMP: matrix metalloproteinase; PDUS: power Doppler ultrasound; PlGF: placental growth factor; RA: rheumatoid arthritis; RF: rheumatoid factor; SDF-1: stromal-cell derived factor-1; SH: synovial hypertrophy; TNFα: tumor necrosis factor alpha; VEGF: vascular endothelial growth factor; VEGFR: vascular endothelial growth factor receptor.

## Competing interests

The authors declare that they have no competing interests.

## Authors’ contributions

JDC had full access to all of the data in the study and takes responsibility for the integrity of the data and the accuracy of data analysis. JDC and RS were responsible for the study design. JR, VR-E, IP, RC, AC, MªVH, JP, JLP, RS, and JDC performed data acquisition, analysis, interpretation, and final approval of the manuscript. Manuscript preparation was by JDC, JR, and JLP. All authors read and approved the final manuscript.
